# Neonatal Multiple Bone Fractures: A Case Report of Hypophosphatasia

**DOI:** 10.1155/crie/9119661

**Published:** 2025-10-30

**Authors:** Doua Khalid Al Homyani, Shahad Khalid Al Hemaiani, Qaydah Qayed Al Harthi, Rayan Al Zahrani, Ali Saad Al Qarni

**Affiliations:** ^1^Division of Endocrinology, Department of Pediatrics, Taif Children's Hospital, Ministry of Health, Taif, Saudi Arabia; ^2^Department of Radiology, King Abdulaziz Specialist Hospital, Ministry of Health, Taif, Saudi Arabia

**Keywords:** asfotase alpha, multiple bone fracture, pediatric, severe perinatal hypophosphatasia

## Abstract

**Background:**

Hypophosphatasia (HPP) is a rare, inherited metabolic bone disorder characterized by mutation in the tissue nonspecific isoenzyme of alkaline phosphatase (ALP) (TNSALP). Perinatal HPP is the most severe type of HPP, primarily characterized by respiratory distress.

**Case Presentation:**

A 2-month-old female infant, born to consanguineous parents with intrauterine limb hypoplasia, sustained a clavicular fracture on day one. She was referred and admitted to the neonatal intensive care unit on day 20 of her life due to the development of seizures and respiratory distress. She presented with short limbs, skeletal hypomineralization, thoracic and pulmonary hypoplasia, hypercalcemia, and ALP levels below the limit of detection (LOD). Severe perinatal HPP was confirmed through genetic analysis. Consequently, she was treated with enzyme replacement therapy (ERT) using asfotase alfa.

**Conclusion:**

Our case emphasized the need for proper diagnosis of severe perinatal HPP to initiate life-saving ERT after delivery. Cooperation between obstetricians and clinical genetics teams is essential to avoid delayed or misdiagnosis.

## 1. Introduction

Hypophosphatasia (HPP) is a rare inherited metabolic bone disorder that can present in perinatal, infantile, childhood, adult form, and odontoid HPP. The incidence of HPP varies around the world. It is reported to be 1 in 500,000 in Europe, 1 in 182,000 in Japan, and 1 in 100,000 in Canada [1, 2]..

HPP is caused by mutations in the alkaline phosphatase (*ALP*) gene, which encodes the tissue-nonspecific isoenzyme of ALP (TNSALP). Since the first mutation was identified by Weiss et al. [3], over 340 mutations have been reported to be responsible for HPP. These mutations lead to a deficiency of TNSALP, resulting in the accumulation of substrates such as inorganic pyrophosphate (PPi), pyridoxal 5′-phosphate (PLP), and phosphoethanolamine (PEA), which are inhibitors of bone mineralization. Elevated levels of PPi, in particular, interfere with the deposition of hydroxyapatite crystals in bones, leading to hypomineralization, fragility, and disrupted bone formation and remodeling. This defect can result in multiple bone fractures, deformities, and other skeletal abnormalities [4, 5].

PLP accumulation affects neurotransmitter metabolism, resulting in vitamin B6-dependent seizures, a frequent complication in severe HPP cases [6]. Additionally, excess PEA is associated with impaired bone mineralization, contributing to the defective formation of bone and exacerbating the characteristic bone fragility [5]. Due to the vast number of TNSALP gene mutations and variable clinical expression, a very wide range of HPP presentations exist.

Depending on onset and symptoms, HPP is categorized into six types: benign prenatal, perinatal lethal, infantile, childhood, adult, and odontoid HPP. Perinatal HPP is the most severe type [7].

The clinical manifestation of perinatal HPP include perinatal growth retardation, skeletal hypoplasia, severe hypercalcemia and hyperphosphatemia, vitamin B6-dependent seizures, early-onset craniosynostosis, and nephrocalcinosis, respiratory distress with apnea, multiple fractures, and markedly shortened limbs [8].

In detail, perinatal HPP often presents with profound skeletal hypoplasia, chest deformities due to rib hypomineralization, and thoracic instability. The hypomineralized ribs and abnormal chest wall mechanics impair lung development, all of which contribute to respiratory distress and pulmonary hypoplasia, resulting in respiratory insufficiency. The condition is further complicated by hypercalcemia, caused by increased bone resorption secondary to impaired bone mineralization, which can also lead to complications such as nephrocalcinosis due to hypercalciuria [9–11].

HPP can be easily misdiagnosed with other clinical conditions having similar manifestations [7]. In the differential diagnosis of patients with low ALP, hypercalcemia, convulsions, and skeletal abnormalities, perinatal HPP should be considered. Since perinatal HPP is fatal in the first months of life due to pulmonary distress, early diagnosis, and proper treatment and management are crucial [12].

Enzyme replacement therapy (ERT) with asfotase alfa, a recombinant human TNSALP, is the only approved treatment for severe perinatal HPP. Asfotase alfa targets mineralization defects by reducing PPi levels and promoting normal bone mineralization. Early initiation of ERT is crucial, as it has been demonstrated to enhance skeletal development, respiratory function, and survival rates.

In addition to ERT, supportive care is essential, including respiratory support for pulmonary hypoplasia, seizure management with vitamin B6, and monitoring of renal function due to the risk of nephrocalcinosis [11].

This is the case report of a baby girl diagnosed with severe HPP in the neonatal period.

## 2. Case Presentation

A 2-month-old female infant, who had limb hypoplasia detected on prenatal ultrasound, was admitted to the hospital at 20 days of age with multiple fractures and abnormal movements. She was delivered by lower-segment cesarean section (CS) (LSCS) secondary to a previous CS at 40 weeks of gestation. The parents were first-degree relatives. This baby was the sixth pregnancy of the mother (G_6_P_5_ + 1), aged 31 years old with two healthy daughters and two other children, died at the age of 8 months and 15 days with a history of multiple bone fractures, refractory seizure, and a history of severe respiratory distress.

At birth, her weight was 2800 g, her head circumference was 32  cm (both below the 3rd percentile), and her length was 45  cm (10th percentile). Her Apgar score was 6 in the first minute and 7 in the fifth minute after birth. At delivery, the female newborn had a clavicular fracture, which was misdiagnosed as osteogenesis imperfecta, and was discharged home.

**Figure 1:**
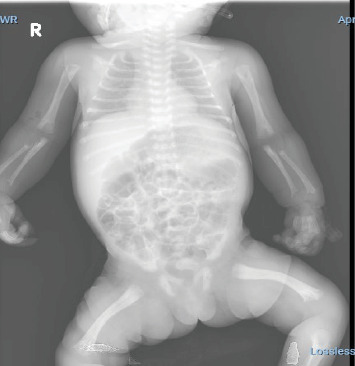
Frontal whole-body radiograph showing skeletal abnormalities. Slender, poorly ossified ribs in the chest, metaphyseal tongues of radiolucency in both upper limbs. Additionally, there is bowing of both femoral bones, absent ossification of the epiphyses of both knees, and metaphyseal tongues of radiolucency involving the distal and proximal tibiae.

**Figure 2:**
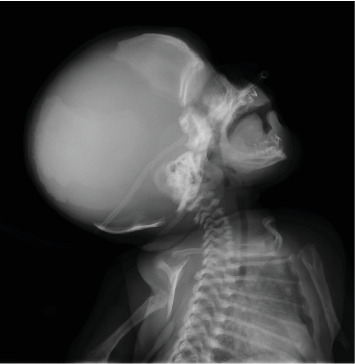
Lateral radiograph of the calvarium. Wide sutures and fontanels.

**Figure 3:**
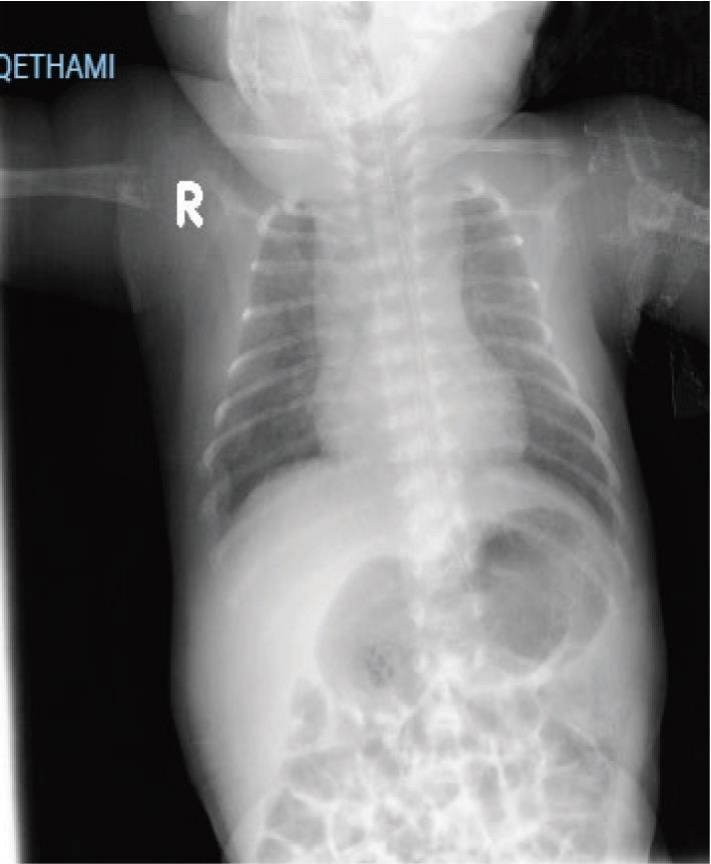
Chest X-ray showing thoracic and pulmonary hypoplasia. Thoracic and pulmonary hypoplasia with thin cortical bone of the ribs.

On day 20 of life, the child was admitted to the NICU with multiple fractures and clonic seizure, which was initially treated with phenobarbitone. The patient was administered vitamin B6 (pyridoxine hydrochloride) at dose of 30 mg/kg/day to control the seizure and was gradually weaned off phenobarbitone. A few days later, she developed respiratory insufficiency and received high-flow oxygen due to respiratory distress caused by pulmonary hypoplasia. Upon physical examination, shortened and bowed arms and legs, widely open fontanels, a short neck, poor muscle tone, and loose joints were observed. The heart examination was normal, and the external genitalia were also normal. The radiological (chest X-ray) examination, as shown in Figure 1. The frontal whole-body radiograph demonstrates slender, poorly ossified ribs in the chest, metaphyseal tongues of radiolucency of both upper limbs. The frontal radiograph of lower extremities demonstrates bowing of both femoral bones, absent ossification of epiphysis of both knee, and metaphyseal tongues of radiolucency involving the distal and proximal tibia (Figure 2). The lateral radiograph of the calvarium shows wide sutures and fontanels.  Figure 3 shows thoracic and pulmonary hypoplasia with thin rib cortical bone. Magnetic resonance imaging (MRI) of the brain was normal.

Baseline biochemical analysis (Table 1) showed hypercalcemia (Ca) 3.18 mmol/L (2.25–2.75), phosphorus: 2.06 mmol/L (1.15–2.15), magnesium: 0.7 mmol/L (0.75–0.95), and serum ALP levels were below the limit of detection (LOD). Vitamin D3 was 85 nmol/L (75–250), while the parathyroid hormone (PTH) 6.70 Pg/mL (12–88 Pg/mL). Urine analysis showed hypercalciuria with an increased calcium creatinine ratio 4.7 mmol/mmol (0.6–0.9). Blood count, as well as liver function, were normal. Renal ultrasound showed bilateral early medullary nephrocalcinosis.

Based on the clinical presentation and biochemical data, severe perinatal HPP was suspected. Autosomal recessive HHP was subsequently confirmed by genetic analysis. *ALPL* gene sequencing was done, and according to the ACMG/AMP guidelines, it is classified as a homozygous likely pathogenic variant, specifically, NM_000478.5:c977G >T p. (Gly326Val) mutations in the *ALPL* gene. This variant has previously been described as a disease – causing mutation in HPP. Thus, the genotype-phenotype correlation can indeed be established, with the Gly326Val mutation serving as a marker for severe phenotypic outcomes in perinatal HPP.

Both parents were found to be heterozygous for the same mutation.

Subsequently, on the 38th day of life, treatment with ERT (asfotase alfa) was initiated after obtaining parental informed consent. The dose was 2 mg/kg, administered subcutaneously three times per week. Following ERT, ALP level was measured as high as 10,238 IU/L (70–350), and serum Ca levels decreased to 2.5 mmol/L. Pyridoxine hydrochloride treatment was continued after the administration of asfotase alfa. Seizures were not observed after starting ERT, and she has remained seizure-free since. At 2 months of age, the patient began to tolerate spontaneous breathing periods with only oxygen supplementation via nasal cannula at 0.25 L/min. Regarding her developmental progress, the baby exhibits overall muscle weakness and hypotonia, but she is beginning to move her limbs more purposefully and responds to sounds and people with smiles.

## 3. Discussion

Here, we report a case of severe perinatal HPP, which is the most fatal form of the disease. The diagnosis of HPP was confirmed through TNSALP mutation analysis. The biochemical, physical, and radiological examination and the genetic analysis of our presented case align with what is described in the literature [4, 13, 14].

As in our case, skeletal findings, such as limb hypoplasia, were the first manifestations detected during the intrauterine period [2]. On her first day of life, she was misdiagnosed with osteogenesis imperfecta due to a clavicular fracture. Two similar case reports were also initially misdiagnosed one with hypoxic-ischemic encephalopathy (HIE) at birth [15], and the other as nutritional rickets at 6 months of age.[16] This misdiagnosis often leads to delayed initiation of therapy and, in some cases, mortality. Since HPP is rare and shares similar manifestations, radiological findings, and biochemical feature with other conditions, many physicians overlook the possibility of HPP during diagnosis [4].

Diagnosing perinatal and infantile HPP is further complicated by its poor prognosis and high mortality rates [9]. In our case, respiratory distress and vitamin B6-dependent seizures were also observed during the first 20 days of life. Pulmonary hypoplasia and chest deformity, as revealed by radiological examination, contributed to impaired respiration, which can result in a 50%–100% mortality rate in most cases of severe perinatal HPP. Additionally, vitamin B6-dependent seizures are usually a fatal prognostic indicator. Nonetheless, a renal ultrasound of our case showed bilateral early medullary nephrocalcinosis as a result of hypercalcemia and hypercalciuria [12].

Therefore, genetic analysis is the definitive confirmatory test for accurately diagnosing HPP. Based on the family history, which includes parental consanguinity and the death of two siblings from undiagnosed HPP, ALPL gene testing was conducted. *ALPL* gene analysis revealed a compound homozygous likely pathogenic variant, specifically, NM_000478.5:(c977G >T) p. (Gly326Val). Parental testing confirmed that both are heterozygous carriers.

In our case, the patient began ERT on day 38. ERT asfotase alfa is the only available therapy for severe perinatal HPP. Before ERT therapy, very few patients survived beyond 1 year, and the majority died within the first few days of life. However, after ERT therapy, 83.3% of documented cases lived and had a significant improvement in their clinical course [17, 18].

## 4. Conclusion

This case report highlights the critical need for early and accurate diagnosis of severe perinatal HPP to ensure timely intervention with ERT using asfotase alfa. The presented case demonstrates the typical challenges associated with diagnosing HPP, including its overlapping symptoms with other conditions, such as osteogenesis imperfecta, and the complexities of managing its severe manifestations, such as hypercalcemia, seizures, and respiratory distress.

Our findings underscore the importance of a multidisciplinary approach involving obstetricians, neonatologists, geneticists, and pediatricians to avoid delays in diagnosis and treatment, which are crucial for survival in severe cases of perinatal HPP. This case reinforces the need for increased vigilance among healthcare providers when encountering neonatal skeletal abnormalities, low ALP levels, and seizures, prompting immediate investigation for possible HPP and consideration of ERT as a life-saving measure.

## Figures and Tables

**Table 1:** Biochemical analysis.

Laboratory parameter	At diagnosis	After 1 month of treatment
Calcium 2.25–2.75 mmol/L	3.18	2.5
Phosphorus 1.15–2.15 mmol/L	2.06	2.27
Magnesium 0.7–0.95 mmol/L	0.7	0.8
ALP 70–350 U/L	0	10,238
PTH 12–88 Pg/ml	6.70	
Creatinine 15–37 Umol/L	39.3	15.6
BUN 2.67–8.07 mmol/L	5.3	2.7
Urine CA0–6.1 mmol/L	2.77	1.8
Urine creatinine 2470–19,200 Umol/L	582	1988
Urine calcium creatinine ratio 0.6–0.9 mmol/mmol	4.7	0.9
Vitamin D75–250 nmol/L	85	98.4
TSH 0.79–5.85 MiU/L	1.41	
FT 47.5–21 Pmol/L	15.1	

## Data Availability

The data that support the findings of this study are available upon request from the corresponding author. The data are not publicly available due to privacy or ethical restrictions.
